# Mucosal T follicular helper cells in SIV-infected rhesus macaques: contributing role of IL-27

**DOI:** 10.1038/s41385-019-0174-0

**Published:** 2019-05-21

**Authors:** Félicien Moukambi, Henintsoa Rabezanahary, Yasmina Fortier, Vasco Rodrigues, Julien Clain, Ghita Benmadid-Laktout, Ouafa Zghidi-Abouzid, Calayselvy Soundaramourty, Mireille Laforge, Jérôme Estaquier

**Affiliations:** 1 Centre de Recherche du CHU de Québec, Université Laval, Québec, G1V 4G2 QC Canada; 2 CNRS FR3636, Université Paris Descartes, 75006 Paris, France

## Abstract

Mesenteric lymph nodes (MLNs), that drain the large and small intestine, are critical sites for the induction of oral tolerance. Although depletion of CD4 T cells in the intestinal lamina propria is a hallmark of HIV infection, CD4 T cell dynamics in MLNs is less known due to the lack of accessibility to these LNs. We demonstrate the early loss of memory CD4 T cells, including T follicular helper cells (Tfh) and a remodeling of MLN architecture in SIV-infected rhesus macaques (RMs). Along with the loss of Tfh cells, we observe the loss of memory B cells and of germinal center B cells. Tfh cells display a Th1 profile with increased levels of the transcription factors that negatively impact on Tfh differentiation and of Stat5 phosphorylation. MLNs of SIV-infected RMs display lower mRNA transcripts encoding for IL-12, IL-23, and IL-35, whereas those coding for IL-27 are not impaired in MLNs. In vitro, IL-27 negatively impacts on Tfh cells and recapitulates the profile observed in SIV-infected RMs. Therefore, early defects of memory CD4 T cells, as well of Tfh cells in MLNs, which play a central role in regulating the mucosal immune response, may have major implications for Aids.

## Introduction

Depletion of peripheral blood CD4 T cells and viral load are key parameters in the follow-up of the disease progression to AIDS. It has been reported that chronic immune T-cell activation and apoptosis correlate with disease progression in human immunodeficiency virus (HIV)-infected humans^1–4^, and in the primate model of pathogenic lentiviral infections.^5–12^ Furthermore, several studies have highlighted the profound loss of CD4 T cells in the gut lamina propria linked with microbial translocation.^13,14^ Mesenteric lymph nodes (MLNs) constitute a specialized lymphoid organ, essential in the genesis of the intestinal immune response, as well as draining the gut-associated lymphoid tissue (GALT). MLNs that are disseminated along the colon and at the base of the thoracic lymphatic duct form the cisterna chyli. In response to the penetration of infectious agents through the intestinal barrier, antigen-presenting cells (APCs) carry microbial antigens via the afferent lymph to MLNs. Therefore, resident T cells in MLNs are kept in a state of “immunological tolerance”, ^15^ through the action of immunosuppressive environmental factors such as TGF-β and IDO1, contributing to the absence of effector CD8 T cells during the simian immunodeficiency virus (SIV) infection.^16^ However, our knowledge of the CD4 T-cell dynamics in MLNs that drain the GALT is limited, due to the non-accessibility of these LNs in HIV-infected individuals.

T follicular helper (Tfh) cells control germinal center (GC) development and are essential to sustaining antiviral antibody production.^17,18^ Tfh cells, which are rare in the blood, produce IL-21^19^, and are specialized providers of T cell help to B cells.^20–23^ Tfh cells selectively express programmed death molecule 1 (PD-1) and CXC chemokine receptor 5 (CXCR5, originally named MDR15/BLR1).^24–26^ Thus, Tfh cells are recruited to lymphoid organs via the follicle-associated chemokine CXCL13/BCA-1 (B cell-attracting chemokine 1).^27,28^ Other groups and ours have reported a defect in Tfh cells during HIV and SIV infections.^29–34^ Thus, HIV-infected individuals with less than 200 CD4 T-cells/mm^3^ show a deficiency in IL-21-secreting CD4 T cells.^35^ Furthermore, higher numbers of Tfh in the peripheral LNs of nonprogressor, compared to progressor SIV-infected rhesus macaques (RMs) have been reported,^36,37^ and splenic Tfh cells are depleted early after SIV infection.^33^ Consistent with such a defect of Tfh cells, impairment in B-cell function occurring early after HIV infection was reported previously.^38–40^ Hence, rapid progression to AIDS is commonly associated with impaired anti-SIV antibodies in RMs,^16^ and depletion of B cells leads to death in SIV-infected Pigtail macaque.^41^ The frequency and quality of Env-specific Tfh cells correlates with the genesis of Env-specific B cells and neutralization.^37^ Cubas et al.^31^ have proposed that excessive and persistent triggering of PD-1 on LN Tfh cells may affect their ability to provide adequate B-cell help.^32^ Thus, Tfh cells are of crucial importance in maintaining efficient B-cell immunity in lymphoid tissues. However, the dynamics of Tfh cells and their relationship with B-cell dynamics in MLNs remain poorly addressed, particularly during the acute phase of infection.

Several transcriptional factors (TFs), including activator and repressor factors, have been reported to play a major role in regulating Tfh cell differentiation.^42–46^ Bcl6 promotes Tfh differentiation, at least in part by suppressing the expression of Tbet (a Th1 TF),^45^ RORγt (Th17),^42^ GATA3 (Th2),^46^ and Blimp-1.^25,47,48^ The TFs, c-Maf and TCF1, have also been reported to be involved in the differentiation and/or function of Tfh cells.^43,44,49–52^ On the other hand, the Krüppel-like factor 2 (KLF2) and Foxo1 restrain Tfh cell differentiation by inhibiting CXCR5 and Bcl6 expression,^53,54^ and regulating the expression of CD62L.^55,56^

Environmental factors, such as cytokines, are critical in regulating Tfh cell differentiation. Whereas IL-6 and IL-21 are essential to induce the expression of Bcl6,^17,57,58^ IL-2, and IL-7 blocked Tfh cell differentiation by inducing STAT5 and T-bet signaling in activated CD4 T cells, or by repressing Bcl6 and CXCR5 expressions.^43,44,59^ IL-12, involved in the generation of Th1 cells, has been reported to induce Tfh differentiation from human naive cells in the presence of IL-6 and IL-21.^60,61^ Furthermore, it has also been shown that individuals deficient for the β chain of the IL-12 receptor have reduced GC responses and Tfh cells.^62^ IL-23, which shares with IL-12 the IL-12Rβ1 chain^63^ induces the expression of CXCR5 and ICOS on human naive CD4 T cells.^64^ An additional member of the IL-12 cytokine family, IL-27, has also been involved in boosting Tfh differentiation in mice^65–67^ but not in human cells^61^ suggesting differences between humans and mice regarding the role of IL-27 in the development of Tfh cells. In the context of an HIV infection, a positive correlation was reported between the levels of IL-27 in the plasma and of proviral DNA in peripheral blood mononuclear cells,^68^ whereas it has been shown that IL-27 inhibits the in vitro HIV infection.^69,70^ Furthermore, other groups as well as ours have previously reported a defect in the expression of mRNAs encoding for p40 and p35 of IL-12.^2,71^ However, the impact of IL-27 on the differentiation of Tfh cells derived from MLNs, in the context of SIV infection, is unknown.

Herein, we have performed the analysis of MLN Tfh cells in SIV-infected RMs in relationship with B-cell dynamics and differentiation. Our results indicate a Th1-like Tfh cell profile and an early loss after an infection associated with the loss of memory B cells. Our results also highlight a dysregulation in the TF network related to the increased levels of inhibitory TFs in Tfh cells, and demonstrates, for the first time to our knowledge, the negative impact of IL-27 on Tfh cell differentiation from MLN. Because MLNs are crucial in maintaining commensal microbiota under control, therefore, the loss of memory CD4 T cells, including Tfh cells, may contribute to the absence of gut immunity favouring microbial translocation during an HIV infection.

## Results

### T- and B-cell dynamics in MLNs of SIV-infected RMs

**Fig. 1:**
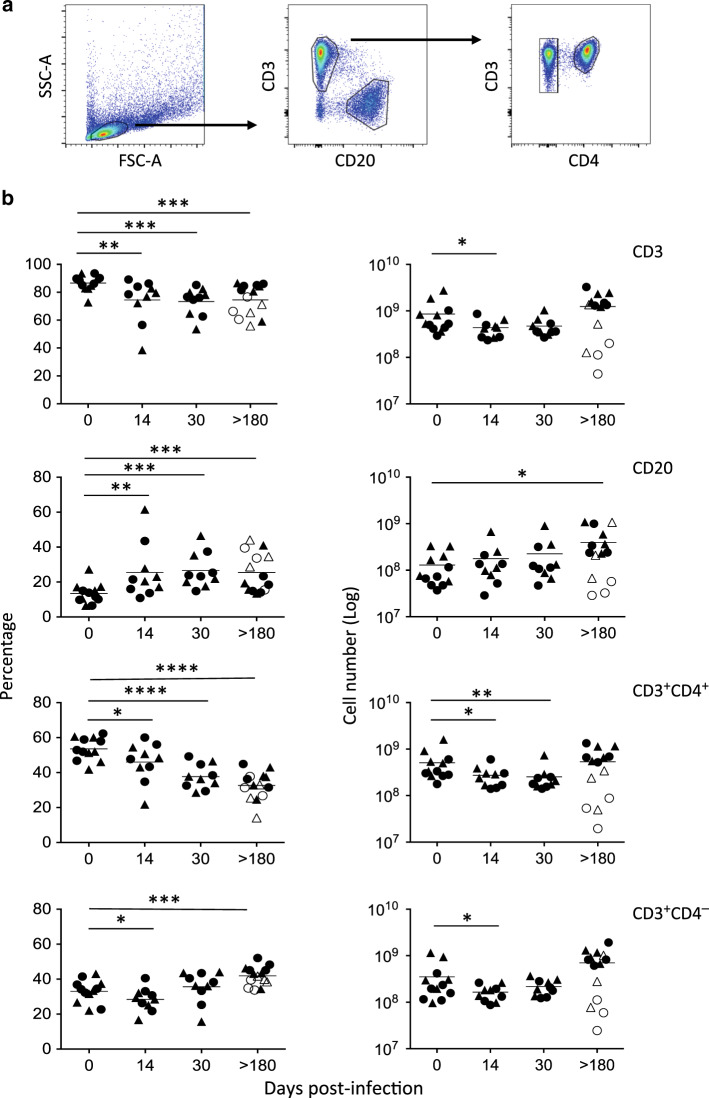
T- and B-cell dynamics in the MLNs of SIV-infected RMs. **a** Gating strategy to identify T and B-cell populations in MLNs by flow cytometry. **b** Histograms show the percentages (left panel) and cell numbers (right panel) of T (CD3^+^, CD3^+^CD4^+^, and CD3^+^CD8^+^) and B (CD3^−^CD20^+^) cells at days 0, 14, 30, and >180 postinfection. Ileocolic MLNs (triangle) and colic MLNs (circle) are indicated in distinct dots for each RM. At day >180, RMs with low levels of CD4 T cells (PB023, PB028, and #1222, empty symbols) and RMs with high levels of CD4 (PB013, PB044, #2012, and #2070R, full symbols) are shown. Statistical analyses were performed using the Mann–Whitney test. *p < 0.05; **p < 0.01; ***p < 0.001; ****p < 0.0001

Our knowledge of T- and B-cell dynamics in MLNs is limited, during the acute phase, due to the limited accessibility to these LNs in HIV-infected individuals. We analyzed their dynamics in uninfected and SIV-infected RMs. The percentages, as well the numbers of CD4, CD8, and B cells in MLNs that drain the upper (ileocolic, Δ) and the lower (colic, O) part of the intestine at different time points postinfection, were assessed (Fig. 1). A significant decrease in the percentage of CD3^+^ T cells (day 0, 86.6% ± 5.7%; day 14, 74.4% ± 15.6%, *p* = 0.0044) was observed, concomitantly with an increase in the percentage of CD20^+^ B cells (day 0, 13.4% ± 5.7% vs. day 14, 25.6% ± 15.6%, *p* = 0.0044) (Fig. 1b) that persists during the chronic phase. The percentages and the numbers of CD4 T cells significantly decreased during the acute phase (day 0, 53.6% ± 6.5%; day 14, 46% ± 11.2%, *p* = 0.0460) (Fig. 1b). In chronically SIV-infected RMs (day > 180), the percentage of CD4 T cells dropped to 32.6% ± 8.1%, whereas in terms of CD4 T cell counts, three RMs (PB023, PB028, and #1222) showed lower numbers, due to a lymphopenia, particularly in colic MLNs (1.95 × 10^7^, 5.3 × 10^7^, and 8.7 × 10^7^, respectively), which is tenfold lower compared to that observed from the other SIV-infected RMs (PB013, 1.3 × 10^9^; PB044, 6.6 × 10^8^; #2012, 5.2 × 10^8^; #2070R, 6.9 × 10^8^). Interestingly, despite lower percentages of CD4 T cells, RMs have even higher numbers of CD4 T cells, due to an increased number of cells recovered from MLNs, compared to healthy RMs (colic MLNs at day 0, 3.2 × 10^8^ ± 1.43 vs. at day > 180, 8.03 × 10^8^ ± 3.65, *p* = 0.0095). Furthermore, our data revealed (PB013, PB044, #2012, and #2070R) higher B-cell counts in RMs in both colic (day 0, 6.4 × 10^7^ ± 2.8 vs. 4.5 10^8^ ± 3.6, *p* = 0.0048) and ileocolic MLNs (day 0, 1.9 × 10^8^ ± 1.1 vs. 5.7 × 10^8^ ± 3.8, *p* = 0.019) (Fig. 1b). Finally, the percentages of CD8 T cells increased at the chronic phase, compared to healthy RMs (day 0, 33.03% ± 6.6%; day > 180, 41.9% ± 5.4%, *p* = 0.0004). Our data revealed that RMs displaying higher CD4 T cell counts also have higher CD8 T-cell numbers in colic MLNs (day 0, 2.05 × 10^8^ ± 1.1 vs. 10 × 10^8^ ± 5.9, *p* = 0.0048) and a trend in ileocolic MLNs (day 0, 4.9 × 10^8^ ± 4.3 vs. 10 × 10^8^ ± 2.8, *p* = 0.057) (Fig. 1b).

**Fig. 2:**
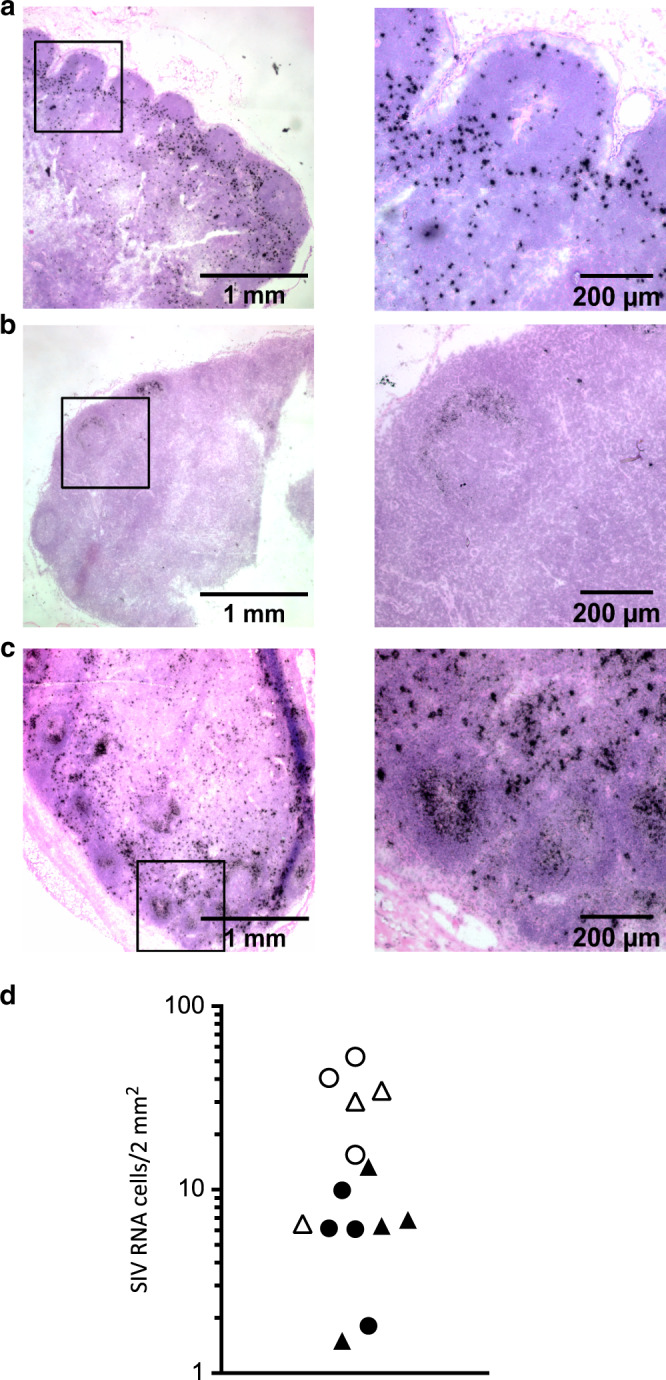
Detection of viral RNA cells in the MLNs of SIV-infected RMs. SIV RNA^+^ cells in LNs are detected by in situ hybridization **a** at day 14 and **b**, **c** in chronically SIV-infected RMs with either high (**b**) or low (**c**) levels of CD4 T cells (Scale bars are included). **d** Ileocolic MLNs (triangle) and colic MLNs (circle) are indicated in distinct dots for each chronically SIV-infected RM (open and closed dots are low and high levels of CD4 T cells, respectively)

Whereas the viral load in PB023 and PB028 reached 10^8^ copies/ml, only 1.5 × 10^6^ copies/ml are detected in RM #1222, which is similar or lower compared to the other RMs such as PB013 or PB044 (3.5 × 10^6^ copies/ml and 5.7 × 10^7^ copies/ml, respectively); RM #2012 showing the lowest, 6.3 × 10^3^ copies/ml (Table 1) suggesting that VL in the blood is not strictly correlated with the extent of CD4 T-cell depletion in MLNs. We have previously reported that MLNs represent a major site for viral replication and stored throughout the course of infection in SIV-infected RMs.^16^ Viral replication in tissues was assessed by in situ hybridization. Diffuse labeling over the follicular dendritic cell (FDC) network in the GC corresponds to virus trapped at the FDC surface (silver grains), whereas individual spots correspond to replicative cells.^16,72^ During the acute phase (day 14) most of the cells are replicative cells (individual spot) (Fig. 2a). In chronically SIV-infected RMs, few SIV RNA cells are detected in RMs with a high level of CD4 T cells in MLNs (SIV RNA cells, 6.4 ± 3.8 cells/mm^2^) and staining corresponds to diffuse labeling in GC (Fig. 2b–d). On the contrary, in RMs having a low level of CD4 T cells, extensive viral replication is detected (SIV RNA cells, 30.1 ± 16.8 cells/mm^2^, *p* < 0.001 compared to the former) (Fig. 2c, d).

**Table 1:** Genotypes of rhesus macaques included in our cohort

Animal OCID	Day of euthanasia postinfection	ΔCD4 counts (cells/mm^3^)	Viral load (copies/ml)	Mamu-A Haplotype 1	Mamu-A Haplotype 2	Mamu-B Haplotype 1	Mamu-B Haplotype 2
PB057	0	323	0.00E+00	A004	A006	B001a	B069a
PB061	0	826	0.00E+00	A006	A008	B001a	B024
PB069	0	1350	0.00E+00	A004	A012	B001a	B056a
9071222	0	1941	0.00E+00	ND	ND	ND	ND
9091442	0	818	0.00E+00	ND	ND	ND	ND
9052732	0	1010	0.00E+00	ND	ND	ND	ND
PB038	14	ND	1.90E+04	A006	A011	B001a	B001a
PB052	14	898	7.90E+04	A002	A008	B069a	B106
PB041	14	−554	8.94E+06	A025	A026	B008	B017f
PB005	14	−784	5.81E+06	A008	A008	B001a	B012a
PB051	14	116	4.67E+07	A004	A004	B012b	B012b
PB049	30	493	9.12E+07	A002	A004	B069a	B069a
PB021	30	−202	3.70E+06	A008	A008	B017a	B055
PB015	30	−492	1.11E+06	A008	A224	B001a	B001a
PB030	30	−954	2.08E+07	A008	A008	B015a	B017a
PB055	30	257	3.24E+07	A008	A019	B017a	B017a
9051222	>180	−1634	1.58E+06	ND	ND	ND	ND
9082012	>180	−1060	6.34E+03	ND	ND	ND	ND
122070R	>180	−505	7.55E+05	ND	ND	ND	ND
PB023	>180	−761	1.39E+08	A001	A006	B001a	B015b
PB028	>180	−668	1.61E+08	A004	A012	B012b	B043a
PB044	>180	−216	5.71E+07	A008	A008	B008	B017a
PB013	>180	−1404	3.57E+06	A001	A008	B002	B055

The table indicates the date of sacrifice, CD4 T cell loss (compared to baseline), and the viral load of uninfected (SIV−) and RMs infected with SIVmac251. Animals were genotyped for MHC class I *Mamu-A* and *Mamu-B* haplotypes. *ND* not determined

Altogether, these results demonstrate the distinct T- and B-cell dynamics occurring in MLNs of SIV-infected RMs in which the extent of SIV RNA cells is associated with lower levels of CD4 T cells in MLNs.

### Early depletion of memory CD4 T cells in MLNs of SIV-infected RMs

**Fig. 3:**
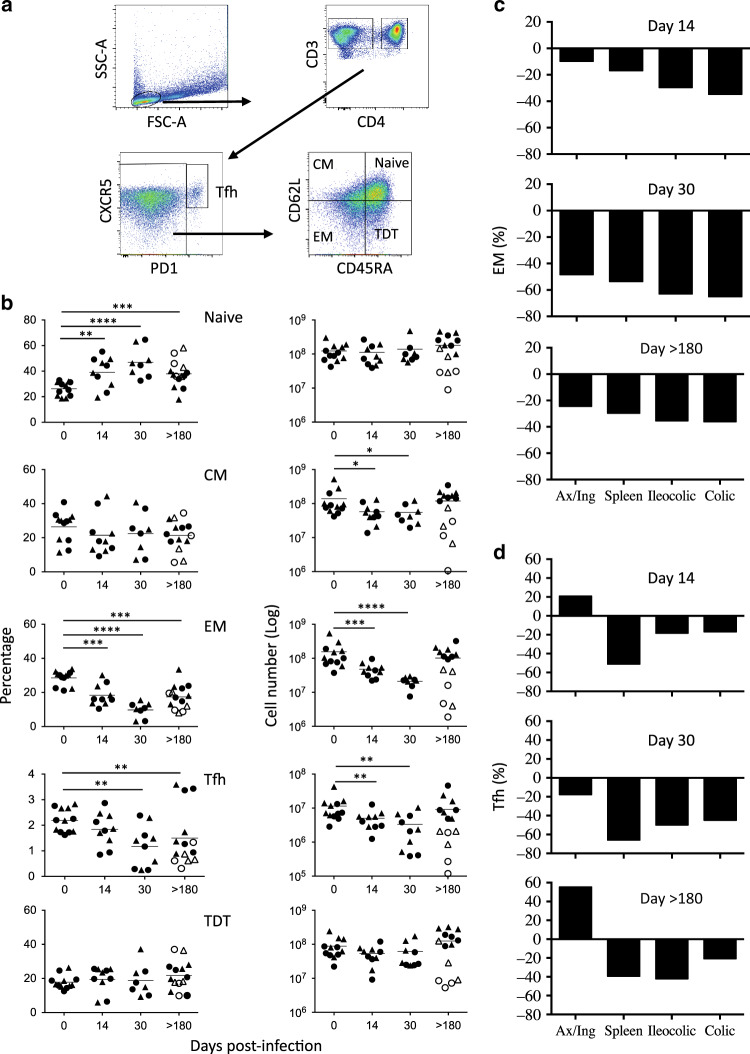
Early depletion of Tfh and of memory CD4 T cells in the MLNs of SIV-infected RMs. **a** Gating strategy to identify CD4 T cell subsets including naive (*N*: CD45RA^+^CD62L^+^), central memory (*CM*: CD45RA^−^CD62L^+^), effector memory (*EM*: CD45RA^−^CD62L^−^), terminally differentiated (*TDT*: CD45RA^+^CD62L^−^) and Tfh (CXCR5^+^PD-1^high^). **b** Percentages (left panel) and cell numbers (right panel) of CD4 T-cell subsets are shown as described in Fig. 1 at days 0, 14, 30, and >180 postinfection. **c**, **d** Histograms show the percentage of decrease in the percentages of EM and Tfh CD4 T-cell subsets compared to healthy RMs at days 14, 30, and >180 postinfection (mean % of EM at day 0 minus the mean % of EM at days postinfection/by the mean % of EM at day 0) × 100. Both axillary/inguinal (Ax/Ing) LNs, ileocolic and colic MLNs, and spleen are shown. Statistical analyses were performed using the Mann–Whitney test. *p < 0.05; **p < 0.01; ***p < 0.001; ****p < 0.0001

We then analyzed the dynamics of the CD4 T cell subsets, including Tfh cells. Cells are defined based on the expression of cell surface markers as effector memory (EM: CD62L^−^CD45RA^−^), central memory (CM: CD62L^+^CD45RA^−^), terminally differentiated (TDT: CD62L^−^CD45RA^+^), naive (CD62L^+^CD45RA^+^), and Tfh cells (CXR5^+^CPD1^high^) (Fig. 3a). Our results demonstrated an early significant increase in the percentages of naive CD4 T cells (day 0, 26.2% ± 5.4%; day 14, 39.1% ± 12%; *p* = 0.0067) (Fig. 3b, left panel). However, their number remained unchanged (Fig. 3b, right panel). On the contrary, we observed a decrease in both the percentage and the number of EM CD4 T cells (day 0, 28.5% ± 4.2%; day 14, 18.4% ± 6.3% *p* = 0.0006) (Fig. 3b, left and right panels). Tfh cells declined in MLNs of SIV-infected RMs at day 30 postinfection (day 0, 2.2% ± 0.5%; day 30, 1.2% ± 0.8%, *p* = 0.0013) (Fig. 3b, left panel). The percentages of CM and TDT were relatively similar in RMs sacrificed at different time points, postinfection. Our data also indicated that the decrease in the percentage of EM compared to healthy RMs in MLNs is similar to the decrease in the percentage observed in the spleen and in Axillary and Inguinal LNs at days 14 and 30 (Fig. 3c), whereas the decrease in the percentage of Tfh cells is higher in the spleen than in MLNs and peripheral LNs (Fig. 3d) suggesting a role of tissue compartmentalization on the early dynamics of EM vs. Tfh cells in SIV-infected RMs

In chronically SIV-infected RMs (day > 180), our results indicated that associated with an increase in the percentage of naïve CD4 T cells, the percentages of both EM and Tfh declined (Fig. 3b, left and right panels). Thus, in SIV-infected RMs (PB023, PB028, and #1222; low level of CD4 T cells), the percentages of EM in colic MLNs (day 0, 27.1 ± 4.2 vs. day > 180, 12.6 ± 5.9, *p* = 0.01) and in ileocolic MLNs (day 0, 29.9 ± 4 vs. day > 180, 13.4 ± 6.1, *p* = 0.01) diminished significantly compared to healthy RMs but despite a trends the difference was not statistically significantly different to the percentage observed in SIV-infected RMs with a high level of CD4 (PB013, PB044, #2012, and #2070R) (19.4 ± 4.3 and 21.8 ± 8.6, respectively). Similarly, the percentages of Tfh in RMs with low level of CD4 (PB023, PB028, and #1222) were lower both in colic (day 0, 2.04 ± 0.43 vs. day > 180, 0.75 ± 0.5, *p* = 0.01) and ileocolic MLNs (day 0, 2.3 ± 0.46 vs. day > 180, 0.7 ± 0.14, *p* = 0.01) compared to healthy RMs and significantly different to the percentages observed in RMs with high level of CD4 (PB013, PB044, #2012, and #2070R) (colic, 2.3 ± 1.4, *p* = 0.05 and ileocolic, 1.89 ± 1.18, *p* = 0.02). Finally, in chronically SIV-infected RMs, we observed a decrease in the percentages of EM in the different tissues analyzed (between 20 and 40% of decrease in the percentage of EM compared to the percentage of EM in healthy RMs) (Fig. 3c), whereas we observed the absence of Tfh decrease in peripheral LNs (axillary and inguinal LNs) or even higher compared to uninfected RMs, but a decreased in MLNs and the spleen (between 20 and 40% of decrease in the percentage of cells) (Fig. 3d).

Due to lymphopenia (Fig. 1), our results indicated that the number of EM and Tfh cells (Fig. 3b) in colic MLNs were tenfold lower in RMs (PB023, PB028, and #1222) compared to uninfected RMs (EM: day 0, 9.1 10^7^ ± 5.1 vs. day > 180, 7.5 10^6^ ± 7.5, p = 0.01 and Tfh: day 0, 6.8 × 10^6^ ± 3.5 vs. day > 180, 7.6 × 10^5^ ± 9.9, *p* = 0.01) as well in ileocolic MLNs (EM: day 0, 2.1 × 10^8^ ± 1.7 vs. day > 180, 3 × 10^7^ ± 2.3, *p* = 0.001 and Tfh: day 0, 1.6 × 10^7^ ± 1.3 vs. day > 180, 1.6 × 10^6^ ± 7.1, *p* = 0.01). In contrary, in RMs with high levels of CD4 (PB013, PB044, #2012, and #2070R), nor the number of EM or the number of Tfh are significantly decreased in colic (EM: 1.6 × 10^8^ ± 1 and Tfh: 1.7 × 10^7^ ± 1.9, *p* = 0.001) and in ileocolic MLNs (EM: 1.7 × 10^8^ ± 3.2 and Tfh: 1.3 × 10^7^ ± 0.8) compared to uninfected RMs.

Altogether, our results indicate a decrease in EM and Tfh cell subsets during the acute phase of the infection, which is more drastic in chronically SIV-infected RMs with low levels of CD4 T cells in MLNs.

### IL-21 and CXCL13 expressions in the MLNs of SIV-infected RMs

**Fig. 4:**
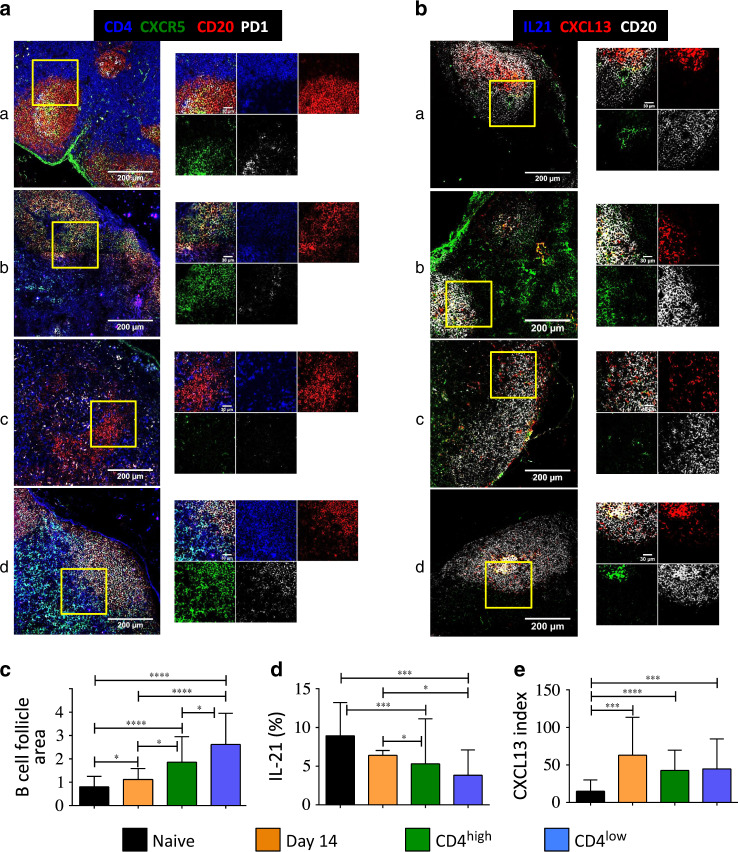
Distribution of Tfh cells in the MLNs of SIV-infected RMs. **a** MLN sections were stained with antibodies against CD4 (blue), CD20 (red), CXCR5 (green) and PD1 (white) and **b** against IL21 (green), CXCL13 (red) and CD20 (white). Tissue sections are imaged by confocal microscopy. Representative pictures of naive a) and SIV-infected RM at days 14 (**b**) and >180 (**c**, **d**) in chronically SIV-infected RMs (CD4^low^ (**c**) vs. CD4^high^ (**d**)). Higher magnification is shown on the right part of the picture. Scale bars are included. **c** B-cell follicle area quantification. Area of B-cell follicles was performed by using Image J software. About 17–26 B-cell follicles were measured for each tissue section, and 3–5 tissue sections were analyzed for each RM. Three RMs were analyzed at each time point. A representative picture of follicle quantification is shown in Supplementary Fig. 1. **d** IL-21 quantification. IL-21 was quantified on the same tissue sections, and the results shown represent the percentage of IL-21 staining per follicle area. Representative picture of IL-21 quantification is shown in Supplementary Fig. 2. **e** CXCL13 distribution within B-cell follicle. CXCL13 distribution in the B-cell follicles was evaluated by the Kurtosis index using Image J software, which is a descriptor of the shape of the biodistribution. A Kurtosis index of 3 is considered as a normal distribution. Representative picture of CXCL13 quantification is shown in Supplementary Fig. 3. Statistical analyses were performed using the Mann–Whitney test. *p < 0.05; **p < 0.01; ***p < 0.001; ****p < 0.0001

Having observed that SIV is present in GC, whereas Tfh cells are diminished, we then assessed by confocal microscopy Tfh cell distribution and its relationship with GC remodeling in the MLNs of SIV-infected RMs. Two weeks after an SIV infection, an enlargement of B-cell follicles can be observed in MLNs (Fig. 4a, c and Supplementary Fig. 1) compared to healthy RMs (Fig. 4a–c). This enlargement of the size increases in chronically SIV-infected RMs (Fig. 4a–c). However, due to the MLN adenopathy, their numbers per mm^2^ of tissue are decreasing. Tfh cells (CD4^+^CXCR5^+^PD-1^+^) are detected in the B-cell follicles of MLNs derived from healthy RMs (Fig. 4Aa), whereas they are hardly detectable in the B-cell follicles in MLNs of RMs with low levels of CD4 T cells (Fig. 4Ac) compared to RMs with high levels of CD4 cells (Fig. 4Ad). IL-21 characterizes Tfh cells. From the same tissue sections, we then assessed the levels of IL-21 in MLNs and observed a lower level of IL-21 in SIV-infected RMs, compared to healthy RMs (Figs 4b–d). The level of IL-21-producing cells was lower in SIV-infected RMs with low CD4 T cells, which is consistent with the depletion of Tfh cells assessed by flow cytometry. T-cell distribution in lymphoid tissues depends in part on the presence of the expressed chemokine. It is well-known that FDC produces CXCL13, the ligand of CXCR5. We consistently found in healthy RMs that CXCL13 is expressed mainly in the GCs of the MLNs (Fig. 4Ba). Our results highlighted changes in the distribution of CXCL13-expressing cells along the B-cell follicle (Fig. 4b) that we quantified by using the kurtosis index (Fig. 4e). Thus, instead of mostly labeling the GCs in healthy RMs, staining is scattered in SIV-infected RMs. Interestingly, this change occurs early after infection (Fig. 4b–e). Moreover, in SIV-infected RMs with low levels of CD4 T cells (Fig. 4Bc), stained cells seem to be more related to perivascular cells.^73^

Altogether, these results demonstrate a remodeling of the MLNs architecture associated with a change in the expression of CXCL13 in SIV-infected RMs with a low level of CD4 with a decline in IL-21 expressing cells.

### Depletion of memory B cells expressing CD95 in the MLNs of SIV-infected RMs

**Fig. 5:**
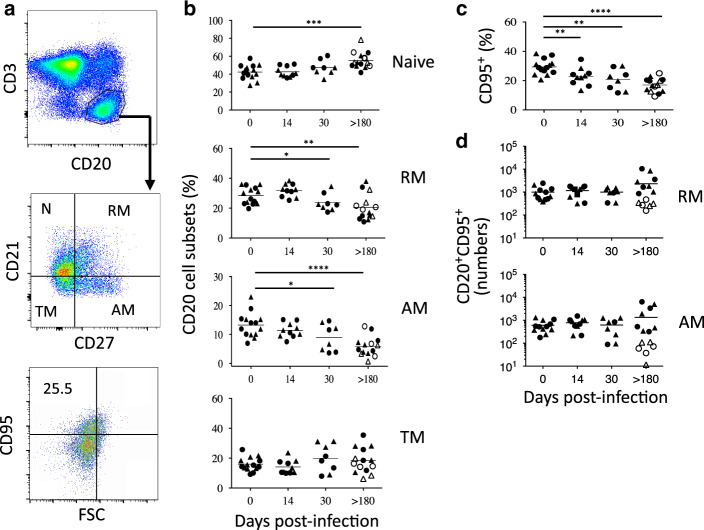
B-cell dynamics in the MLNs of SIV-infected RMs. **a** Gating strategy to identify B-cell subsets, including naive (N: CD21^+^CD27^−^), resting memory (RM: CD21^+^CD27^+^), activated memory (AM: CD21^−^CD27^+^) and tissue memory (TM: CD21^−^CD27^−^) B cells. Furthermore, the expression of Fas (CD95) was assessed by flow cytometry. **b** Percentages of B-cell subsets are shown at days 0, 14, 30, and >180 postinfection, as described in Fig. 1. **c** Histograms show the percentage of B cells expressing CD95. **d** B-cell numbers of RM and AM subsets expressing Fas (CD95) are shown as described in Fig. 1. Statistical analyses were performed using the Mann–Whitney test. *p < 0.05; **p < 0.01; ***p < 0.001; ****p < 0.0001

**Fig. 6:**
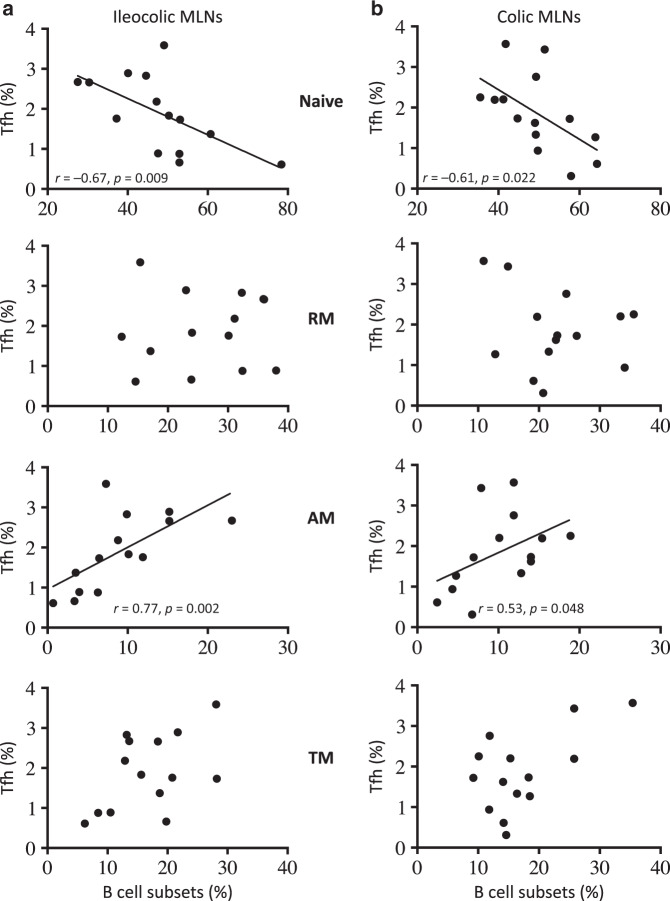
Correlation between Tfh cells and memory B cells. Tfh were plotted against B-cell subsets in either ileocolic or colic MLNs. Each dot represents an individual RM at the time of death. Spearman analysis was used for correlations. The *r* and *p* values are indicated in the figures

Because we observed lower Tfh cell numbers in the MLNs of SIV-infected RMs, which are considered to be essential for B-cell differentiation with a CXCL13 staining, which is generally observed during late embryonic splenic vasculature in which B cells are essentially immature,^73^ we analyzed the B-cell differentiation. B-cell subsets are defined as follow: naive (CD21^+^CD27^−^), resting memory (RM: CD21^+^CD27^+^), activated memory (AM: CD21^−^CD27^+^), and tissue memory B cells (TM: CD21^−^CD27^−^) (Fig. 5a). It was previously reported that in healthy RMs, the relative frequencies of B-cell subsets differed substantially from blood to peripheral LNs, and upon infection, a significant decrease in the frequency of naive B cells from peripheral LNs is compensated by an increase in CD27^+^ memory B cells.^74^ In MLNs, no major changes were observed in the distribution of B-cell subsets during the acute phase of infection (Fig. 5b). At day 30 postinfection, we observed that the percentages of AM (CD21^−^CD27^+^) and RM (CD21^+^CD27^+^) B cells in MLNs are slightly decreased compared to the percentage of B cells from healthy RMs (AM: day 0, 13.4% ± 4.2%; day 30, 8.9% ± 4.6% *p* = 0.039, and RM: day 0, 28.4% ± 5.7%; day 30, 23.7% ± 5.8%, *p* = 0.01) (Fig. 5b). In chronically SIV-infected RMs (>180 days), these percentages of AM and RM B cells were lowered reaching 5.9% ± 3.4% (*p* < 0.001) and 20.5% ± 8.8% (*p* = 0.004), respectively (Fig. 5b). Conversely, the percentage of naive B cells increased (day 0, 42.4% ± 8.2%; day 180, 55.2% ± 9.2%, *p* < 0.001). Because, as shown in Fig. 1b, our data indicated that the percentages of B cells increase at day 180 in MLNs (Fig. 1b), we reevaluated these percentages accordingly. Thus, the percentage of naive B cells increased, particularly in RMs (PB023, PB028, and #1222) with low levels of CD4 (19.7% ± 9.1), whereas this percentage was only 10.4% ± 4.2% (*p* < 0.02) in RMs with high levels of CD4 (PB013, PB044, #2012, and #2070R). Thus, the fraction of naive B cells in MLNs increased in the former. The percentages in the other B-cell subsets were not significantly different between both groups of RMs. We then assessed the correlation between Tfh and B-cell subsets in colic and ileocolic MLNs (Fig. 6). We observed a negative correlation between the percentages of naive B cells and Tfh cells, and a positive correlation with AM B cells (Fig. 6). We also evaluated Fas (CD95) expression, which is highly expressed on GC B cells,^75,76^ and contributes to the regulation of B cells.^77,78^ We observed a significant decrease in the percentage of B cells expressing Fas during the acute (day 0, 16.1% ± 2.9%; day 14, 12.6% ± 5.6% *p* = 0.0126) and the chronic phases of SIV infection (day > 180, 7.1% ± 2.2% *p* = 0.0001) (Fig. 5c), indicating the decrease of GC B cells in SIV-infected RMs. Fas is mainly expressed on RM (49.3% ± 9.4%) and AM B cells (72.5% ± 10.2%) in healthy RMs, whereas only 15.6% ± 4.8% of TM expressed Fas, and less than 5% of naive cells. At the chronic phase, these percentages decreased to 40.9% ± 14.2% (*p* = 0.03) and 58.5% ± 17.9% (*p* = 0.01) for RM and AM B cells, respectively. By analyzing the pool of B cells expressing CD95 (Fig. 5d), we found that the numbers of RM and AM B cells expressing Fas are reduced in PB023, PB028, and #1222 (RM B cells, 301 ± 105 vs. 998 ± 667 in healthy RMs, *p* = 0.0002; AM B cells, 66.7 ± 39.3 vs. 610 ± 353 in healthy RMs, *p* < 0.0001), whereas these numbers were higher in RM B cells of PB013, PB044, #2012, and #2070R (3841 ± 364 vs. 998 ± 667 healthy RMs, *p* = 0.002) and not significantly different for AM B cells (2299 ± 2380 vs. 610 ± 353, *p* = ns).

Altogether, our results strongly suggested that AM and GC B cells are mostly affected by the absence of Tfh cells, and their depletion characterized animals having low CD4 T cells in MLNs.

### Commutation of effector memory MLN Tfh cells toward central memory is associated with T-bet expression and Stat5 phosphorylation in SIV-infected RMs

**Fig. 7:**
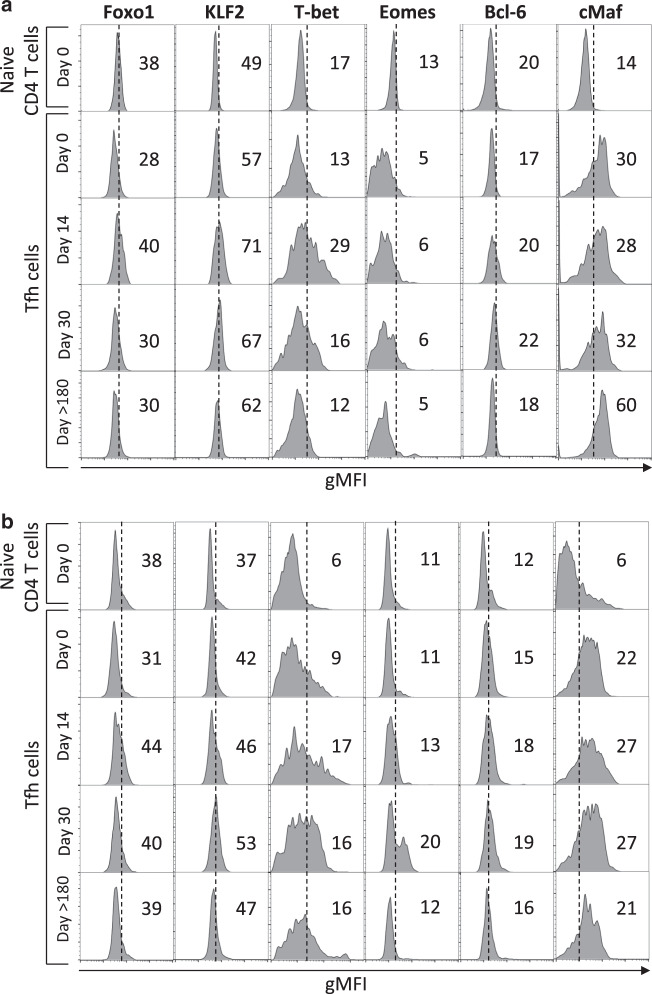
Expression of transcriptional factors in the Tfh cells of SIV-infected RMs. **a,** **b** Histograms show the expression profile of Foxo1, KLF2, T-bet, Eomes, and c-Maf in Tfh cells of **a** Ileocolic and **b** colic MLNs from healthy (day 0) and SIV-infected RMs at days 14, 30, and >180 compared to naive CD4 T cells of a healthy RM and performed the same day for comparison. The histograms shown are representative of three independent experiments. Mean fluorescence intensities are indicated

**Fig. 8:**
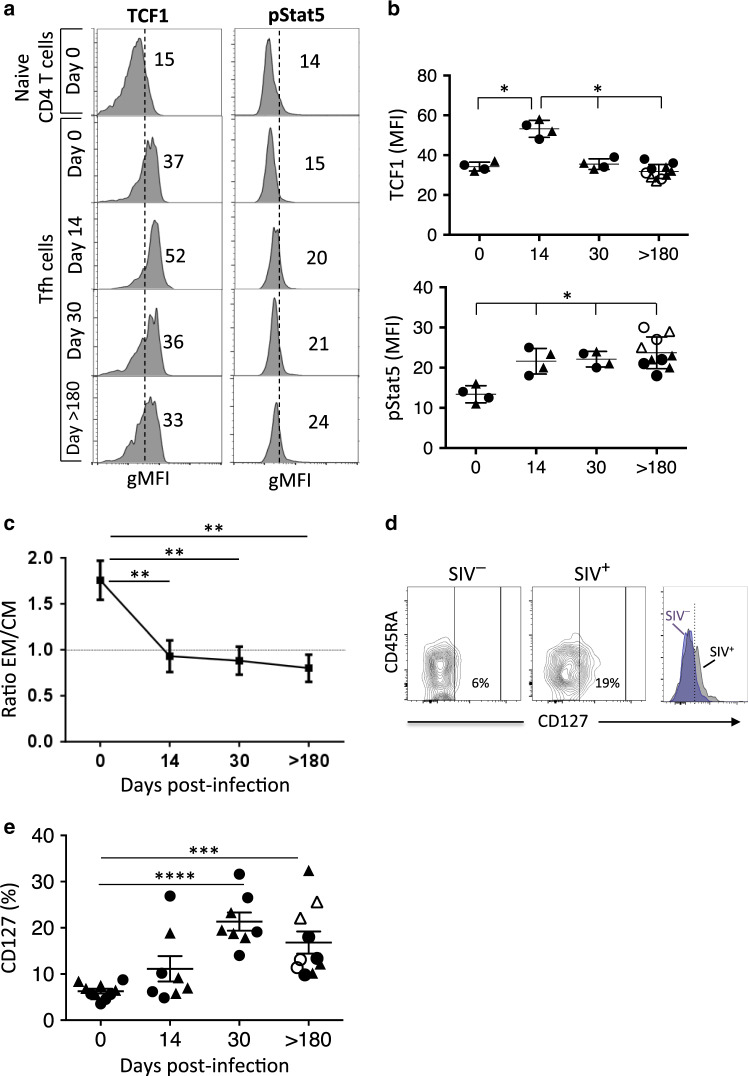
Expression of TCF1, pStat5, and CD127 in the Tfh cells of SIV-infected RMs. **a** Expression profile of pStat5 and TCF1 in Tfh cells of colic MLNs from healthy (day 0) and SIV-infected RMs at days 14, 30, and >180 in comparison to naive CD4 T cells from a healthy RM. Mean fluorescence intensities are indicated. **b** Histograms show the expression of TCF1 and pStat5 in Tfh cells from ileocolic MLNs (triangle) and colic MLNs (circle) are indicated for each RM. At day > 180, CD4^high^ (closed symbol) and CD4^low^ (open symbol) are indicated. **c** Histogram shows the ratio of effector memory (CDR45^−^CD62L^−^) vs. central memory (CDR45^-^CD62L^+^) Tfh in MLNs of SIV-infected RMs at days 0, 14, 30, and >180 postinfection. Data are the mean ± SEM of four individuals at each time point. **d** Representative dot plots depicting the expression of CD127 on Tfh cells in uninfected (SIV^−^) and SIV-infected RMs (SIV^+^). Overlay is shown on the right part. **d** Histograms show the percentage of Tfh cells expressing CD127 at days 0, 14, 30, and >180 postinfection. Ileocolic MLNs (triangle) and colic MLNs (circle) are indicated for each RM. At day > 180, CD4^high^ (closed symbol) and CD4^low^ (open symbol) are indicated. Statistical analyses were performed using the Mann–Whitney test. *p < 0.05; **p < 0.01; ***p < 0.001; ****p < 0.0001

Several TFs, including activator factors such as Bcl6, c-Maf, and TCF-1,^42–46,49–52^ and repressor TFs, such as KLF2, Foxo1, and T-bet^53,54^ have been reported to play a major role in regulating Tfh cell differentiation. Our results demonstrated a transient increase in the expression of KLF2 and Foxo1 at days 14 and 30, along with an increase in the expression level of T-bet in both colic and ileocolic MLNs (Fig. 7a, b). Bcl6 is slightly increased at day 30, whereas cMaf expression increased at day > 180 only in colic MLNs. In addition to cMaf, TCF1, which is essential for both the initiation of differentiation and the effector function of Tfh cells, is increased only at day 14 post-infection (Fig. 8a, b). Because KLF2 and Foxo1 regulate the expression of CD62L,^55,56^ we measured the ratio of effector memory (CD62L^−^CD45RA^−^) vs. central memory (CD62L^+^CD45RA^−^) phenotype. Our results indicated that Tfh cells switch from an effector memory phenotype in healthy RMs (*r* = 1.8 at day 0) to a central memory phenotype after SIV infection (at day 14, *r* = 0.93; at day 30, *r* = 0.88) (Fig. 8c). Thus, our results suggest that early after infection, inhibitory TFs are increased that may impact on Tfh differentiation.

We then analyzed the phosphorylation status of Stat5, which has been associated with a block in the differentiation of Tfh cells.^43,44^ Interestingly, early after SIV infection and thereafter, Stat5 phosphorylation increases and remains elevated in Tfh cells (Fig. 8a, b). Because IL-7 induces Stat5 phosphorylation and has been reported to repress Tfh cell differentiation,^59^ we investigated the cell surface expression of IL-7 receptor (CD127) on Tfh cells (Fig. 8d, e). Our results revealed a significant and progressive increase in the percentage of Tfh cells expressing CD127 in MLNs during the acute (day 0, 6.3% ± 1.2%; day 30, 21.3% ± 5.6%; *p* < 0.0001) and the chronic phases of SIV infection (day > 180, 18.3% ± 7.8%; *p* < 0.0001) (Fig. 8e).

Altogether, our results demonstrate that Tfh cells from MLNs are associated with a dysregulation of the TF network early after SIV infection, switching from EM to CM associated with Stat5 phosphorylation.

### SIV infection leads to the downregulation of mRNAs coding for the members of the IL-12 cytokine family

**Fig. 9:**
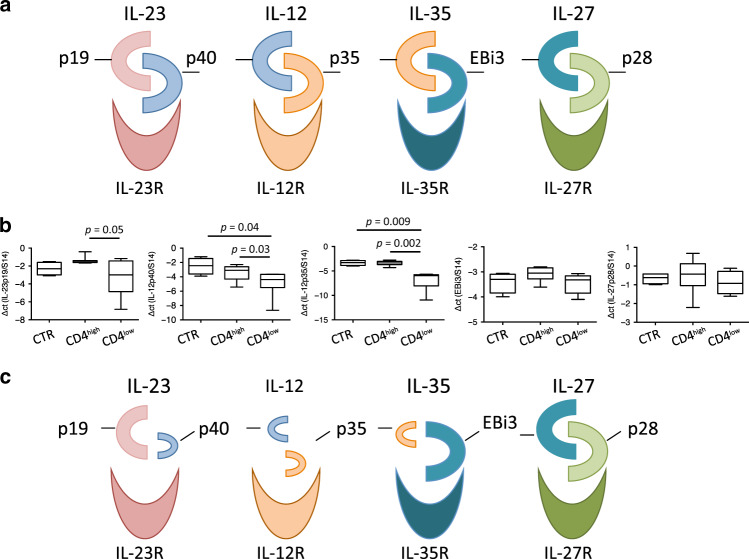
mRNA expression of the IL-12 cytokine family members in the MLNs of SIV-infected RMs. **a** Schematic representation of the IL-12 cytokine family, including IL-23, IL-12, IL-35, and IL-27. **b** mRNA expressions of the α and β chains of IL-12 family members in the MLNs of healthy (*n* = 4), CD4^high^ (*n* = 4) and CD4^low^ (*n* = 3). Statistical analyses were performed using the Mann–Whitney test. **c** Schematic representation of the IL-12 cytokine family in MLNs of SIV-infected RMs in which the levels of CD4 is low

Several groups have reported that IL-12, a cytokine driving the Th1 response, induces IL-21 expression in human CD4 T cells,^60,61^ whereas the impact of IL-27 on Tfh cells seems to be species-dependent.^61,65–67^ Formation of IL-12 is related to mRNA expression coding for the p40 β chain and the p35 α chain, whereas IL-27 is formed by the association of the Ebi3 β chain with the p28 α chain. The p40 β chain can also pair with the p19 α chain forming the IL-23, while the Ebi3 β chain with the p35 α chains can form IL-35^79^ demonstrating the complexity of this family of interleukins (Fig. 9a). In the context of HIV-infected individuals, other groups and ours have previously reported a defect in the p40 and p35 mRNAs forming the IL-12.^2,71,80^ We thus decided to quantify the relative levels of mRNA expression coding for these different α and β chains that form the IL-12 cytokine family. Indeed, the protein-detecting tools (antibodies and ELISA), particularly IL-27 and IL-35, do not cross-react for monkeys. Consistent with our previous results, we observed lower levels of the mRNA encoding for p40 and p35 in MLNs of RMs with low levels of CD4 cells compared to healthy RMs (p40, −4.6 ± 0.64 vs. −2.5 ± 0.56, *p* = 0.02 and p35, −6.5 ± 0.65 vs. −3.3 ± 0.26, *p* = 0.001, respectively) (Fig. 9b). Our results also indicated a lower level of the p19 mRNA between both groups of SIV-infected RMs (p19, −3.1 ± 0.72 vs. −1.4 ± 0.10, *p* = 0.05). On the contrary, no significant difference was observed in the levels of mRNA coding for EBi3 and p28 mRNA in SIV-infected RMs compared to healthy RMs (Fig. 9b). These results indicated that SIV infection is associated with lower transcripts that form IL-12, IL-23, and IL-35 in the MLNs of monkeys with low levels of CD4 T cells, compared to healthy or RMs with high levels of CD4 T cells (Fig. 9c).

Thus, our results indicate a major decline in the expression of several transcripts required to form several IL-12 family members in MLNs, but not for those coding for IL-27.

### IL-27 induces inhibitory TFs and T-bet expression in Tfh cells from the MLNs of RMs

**Fig. 10:**
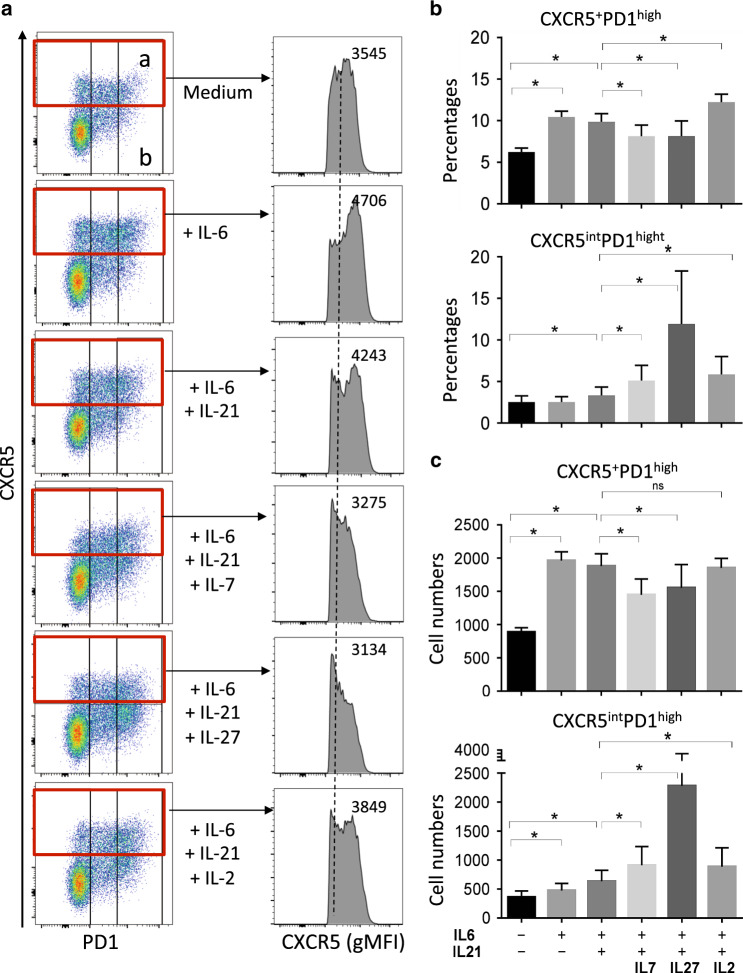
Impact of IL-27 on Tfh cells derived from MLNs. (**a**, left panel) Dot plots depicting the expression of CXCR5 and PD1 after stimulation of MLN cell suspension isolated from a healthy RM with coated CD3 and CD28 mAbs (0.1 and 0.25 μg/ml, respectively) in the absence (medium) or presence of IL-6, IL-21, IL-7, IL-27, and IL-2. (**a**, right panel) Histograms show the fluorescence intensity of CD4 T cells expressing CXCR5 (gMFI) gating on the region depicted in red. **b**, **c** Histograms show the percentages and cell numbers of CXCR5^+^PD1^high^ (region a) and CXCR5^low^PD1^high^ (region b). Data are the mean ± SEM of three independent experiments. Statistical analyses were performed using the Mann–Whitney test. *p < 0.05

It has been reported that IL-6 is essential to induce IL-21 and sustain Tfh cells.^81–83^ The IL-6 receptor is formed by the association with the IL-6 receptor and the gp130. This latter is shared with the IL-27 receptor^84^ involving the WSX-1/T (homologous to the IL-12Rβ2 subunit) suggesting that IL-6 and IL-27 may be competitive for gp130. Therefore, we decided to evaluate the impact of IL-27 on Tfh cells derived from MLNs in comparison to IL-2 and IL-7 reported to antagonize Tfh cell differentiation through Stat5 phosphorylation.^60,61^ Because Tfh cells are subsequently lost in the absence of B cells,^85^ we used MLN cell suspension instead of purified T cells. MLNs are stimulated in the presence of low concentrations of coated CD3 and CD28 mAbs and incubated in the absence or presence of IL-6 and IL-21 to support the growth of Tfh cells, and in the absence or presence of IL-2, IL-7, or IL-27. Whereas the mean fluorescence intensity of CXCR5 is increased at day 5 in the presence of either IL6 or IL-6/IL-21, our results indicated that IL-2, IL-7, and IL-27 downregulated CXCR5 expression (Fig. 10a). IL-6/IL-21 increased the percentage and the number of Tfh cells (CXCR5^+^PD1^high^) compared to T-cell stimulation alone (Fig. 10b, c). On the contrary, IL-7 or IL-27 decreased both the percentage and the number (Fig. 10b). Our results highlighted that IL-27 increases the percentage (Fig. 8b) and the number (Fig. 10c) of the CXCR5^low^PD1^high^ population.

**Fig. 11:**
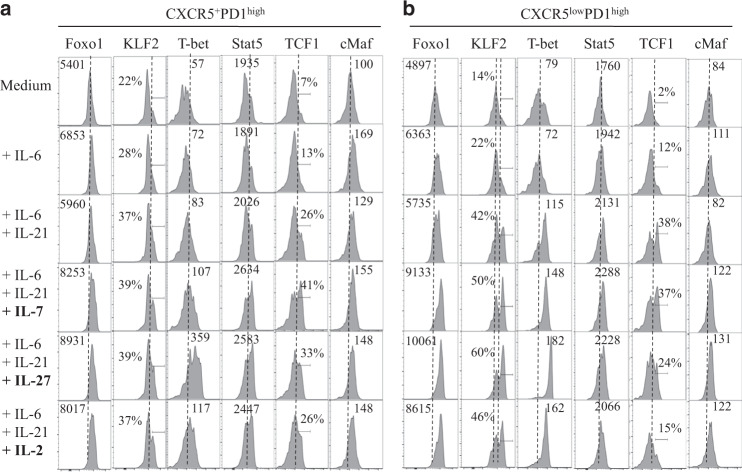
Impact of IL-27 on the expression of transcriptional factors in Tfh cells. Histograms show the expression profile of Foxo1, KLF2, pStat5, TFC1, and c-Maf in CXCR5^+^PD1^high^ (**a**, area a in Fig. 10) and CXCR5^low^PD1^high^ (**b**, area b in Fig. 10) subsets. Thus, data are representative of three independent experiments

We then analyzed in more detail the impact of interleukins on the levels of TFs in CXCR5^+^PD1^high^ (Fig. 11a). Because the expression of CXCR5 is downregulated we also extended the analysis of TFs expressions in CXCR5^low^PD1^high^ (Fig. 11b). In comparison to cells stimulated with CD3/CD28 only, IL-6 increased the percentage of cells expressing TCF1 and the levels of cMaf in CXCR5^+^PD1^high^ T cells consistent with the increase in Tfh cells (Fig. 11a). The addition of IL-21 with IL-6 increased TCF1 but also KLF2. The levels of the two TFs, Foxo1, and T-bet, in CXCR5^+^PD1^high^ cells are increased in the presence of IL-2 and IL-7 that is associated with Stat5 phosphorylation (pStat5) compared to IL-6/IL-21 (Fig. 11a). Most importantly, our results highlighted that IL-27 upregulates the expression of Foxo1, T-bet, and pStat5 in CXCR5^+^PD1^high^ cells (Fig. 11a). IL-27 was more efficient to increase the level of T-bet expression than that observed in the presence of IL-2 or IL-7. However, the expression of KLF2 is not modulated in CXCR5^+^PD1^high^ cells compared to IL-6/IL-21 (Fig. 11a). These results demonstrated that IL-27 is a potent cytokine in inducing a Th1 profile in this Tfh cell subset. Our results also highlighted that IL-2, IL-7, and IL-27 enhanced the levels of Foxo1 and T-bet in CXCR5^low^PD1^high^ cells (Fig. 11b) as well of KLF2. IL-2 and IL-27 induced the downregulation of TCF1 compared to IL-6/IL-21 in CXCR5^low^PD1^high^ cells (Fig. 11b).

Altogether, our results highlighted that IL-27 not only induces the expression of TFs, which are inhibitory of Tfh cell differentiation but is also more potent than IL-2 or IL-7 in inhibiting mucosal Tfh cells.

## Discussion

In this study, we demonstrated that IL-27, a member of the IL-12 cytokine family, is critical to promote in vitro Th1-like Tfh cells from MLNs, and to induce the expression of inhibitory TFs and Stat5 phosphorylation reported to negatively regulate Tfh cell differentiation. Tfh cells derived from MLNs of SIV-infected RMs display a similar profile early after infection. Our results also highlighted in RMs with low levels of CD4 T cells in MLNs with mRNAs coding for the α and β chains forming the IL-12, IL-23, and IL-35 are decreased, whereas those forming the IL-27, EBi3, and p28 are unchanged. Furthermore, our results indicated the loss of mucosal Tfh cells along with the loss of memory B cells and GC B cells expressing CD95 in SIV-infected RMs. Because MLNs are critical components of the GALT and the largest in the body, maintaining oral tolerance,^86–88^ this early abnormal differentiation and depletion of CD4 T cells in these inductive sites (MLNs) could provide a rationale to the absence of replenishment of these effector cells in the lamina propria, the main effector sites, which therefore may contribute to the inability of the immune system to control HIV and SIV infections in mucosal tissues.^9,10,14,89–92^

During LCMV infection in mice, it was been reported that IL-27 induced IL-21 from CD4 T cells, whereas IL-6 is required to sustain Tfh cells.^66,82^ In particular, FDCs produce IL-6, supporting GC reactions during immunization^66,82,93^ consistent with the observation that the absence of IL-6 is associated with an early defect in Tfh cells.^81^ Whereas IL-27 induces in vitro IL-21 secretion from murine naive T cells,^65,67^ IL-27 was fourfold less potent than IL-12 to induce IL-21, and IL-27 had little effect on Bcl-6 mRNA expression, suggesting that IL-27 did not induce full Tfh differentiation.^65^ Other studies indicate that only IL-12, and not IL-27, induced IL-21 expression from human cells.^61^ Thus, the impact of IL-27 in the genesis of Tfh cells was proposed to be species-dependent,^61^ which may have considerable implications for B-cell vaccine development in which Tfh cells are critical. Our results in monkeys demonstrated that in vitro IL-6, in combination with IL-21, increases Tfh cells, whereas IL-27 impacts on this pool by downregulating the expression of CXCR5, similarly as IL-2 and IL-7. Our results also indicated that IL-27 increases T-bet, which is consistent with earlier reports^94,95^ but also induces Stat5 phosphorylation, similarly to IL-2 and IL-7 reported to negatively impact Tfh function.^43,44,49,59,78^ Importantly, we observed that Tfh cells from SIV-infected RMs display higher levels of Stat5 phosphorylation. Although we did not modulate in vivo IL-27 in SIV-infected RMs due to the actual absence of cross-reacting reagents such as neutralizing antibody, our results performed in vitro and the phenotype associated with Tfh cells derived from MLNs of SIV-infected RMs strongly suggested that in the absence of the IL-12, IL-27 may contribute to the impairment of mucosal Tfh cells during SIV infection.

For the first time, to our knowledge, our results also highlighted that IL-27 modulates the expression of critical TFs, namely KLF2 and Foxo1, which have been reported to inhibit CXCR5 and CD62L expressions.^53,54^ Herein, we found that Foxo1 is increased in the Tfh cells of MLNs, and a switch for an effector memory to a central memory profile (CD45RA^−^CD62L^+^) is observed. Furthermore, our results indicate that IL-27 inhibits the expression of TCF1 in vitro, which is induced by IL-6/IL-21 and contributes to the differentiation of Tfh cells.^58,96^ We observed that the expression of TCF1 is transiently expressed (day 14) in the Tfh cells of SIV-infected RMs. Therefore, once again for the first time as far as we know, we established the TF networking associated with IL-27 in Tfh cells. Thus, our results indicate that IL-27 in monkeys may contribute to the genesis of Th1-like Tfh cell population by impairing CXCR5 expression and inducing inhibitory TFs that may have an impact on the distribution and function of Tfh cells in GCs during SIV infection. However, we cannot exclude the role of additional cytokines in regulating mucosal Tfh cells in SIV-infected RMs. Indeed, our results indicated that in vitro IL-7 also decreases CXCR5 expression. Interestingly, whereas Tfh cells express low levels of CD127 in healthy RMs, a sustained increase in the expression of CD127 at the cell surface of Tfh cells in MLNs of SIV-infected RMs was observed. Therefore, in a context in which IL-7 has been reported to be increased in the lymphoid tissues and plasma of HIV-1-infected individuals,^97–99^ IL-7 may also contribute to the inhibition of Tfh cell differentiation in the MLNs of SIV-infected RMs. Although we identified a TF network associated with Tfh differentiation during SIV infection, this cannot exclude the role of additional TFs reported to contribute to their regulation such as the transcription factor achaete-scute homolog 2 (Ascl2) as well as the inhibitor of DNA-binding 2 (Id2) and Id3^100,101^ demonstrating the complexity of Tfh regulation in the context of infectious diseases. Interestingly, several chronic infectious diseases such as malaria and leishmania have also been reported to be associated with the abnormal differentiation and redistribution of Tfh cells in lymphoid tissues,^102,103^ but the role of IL-27 in regulating Tfh cells has not been addressed in each of these models.

Tfh cells are essential to the regulation of GC size, B-cell differentiation, and B-cell Ig affinity maturation.^17,18,20^ Herein, we reported that the percentages of AM (CD21^−^CD27^+^) and RM (CD21^+^C27^+^) B cells declined in the MLNs of chronically SIV-infected RMs, and a positive correlation between Tfh cells and AM B cells was observed. Furthermore, GC B cells that expressed Fas (CD95) progressively declined. In particular, AM B cells expressing CD95 are lowered in SIV-infected RMs with low level of CD4 T cells compared to RMs having high level of CD4 T cells in MLNs. It is well-known that CD95 contributes to the regulation of memory B cells.^75–78^ The co-engagement of CD40 and the antigen-receptor protect B cells from CD95 ligation-mediated cell death^104,105^ through NF-kB activation.^106^ Therefore, the loss of Tfh cells may lead to the absence of co-stimulatory molecules that are essential for B-cell survival and differentiation leading to the depletion of B cells expressing CD95 in MLNs. Earlier reports have indicated that dying B cells are localized in B-cell areas surrounding GCs where Tfh are normally localized, leading to a polyclonal B-cell activation in intestine and GC involution in HIV-infected individuals, early after infection.^107^ Although we did not directly investigate the specificity of Tfh cells in MLNs, the precocity of Tfh depletion argues against a SIV specificity of these Tfh cells and supported the idea of a more global defect that may have a major impact on the quality of the immune repertoire. In this context, it has been reported that the frequency and quality of Env-specific Tfh cells correlates with the genesis of Env-specific B cells and neutralization and negatively correlates with the expression of T-bet.^37^ An enrichment of abnormal B-cell subsets was reported in viremic patients^108^ that is restored by early antiretroviral therapy^109^ indicating that a defect occurs early after the moment of infection.^38–40^ In the spleen of SIV-infected RMs, we also recently reported an early defect in the differentiation of B cells.^33^ A loss of B cells and an absence of seroconversion was also reported in monkeys, which rapidly progressed to AIDS.^110^ The comparison of pathogenic and nonpathogenic monkeys has also indicated a correlation between the levels of CD4 T cell depletion and the capacity to generate an IgG response.^11,72^ Therefore, the depletion and the profile of Tfh cells derived from SIV-infected RMs may contribute to the general observation that AIDS is associated with the phenotypic and functional abnormalities of B cells that arise over the course of the infection.

Petrovas et al.^34^ have reported that preserved LNs architecture in chronically SIV-infected RMs is associated with increased numbers of Tfh cells. Other groups indicated the loss of Tfh cells in progressor, compared to nonprogressor SIV-infected RMs,^36,37^ and monkeys progressing faster to AIDS exhibit the loss of IL-21-secreting cells associated with involution of the GCs.^111^ Herein, we observed lower level of IL-21-secreting cells in MLN follicles, which is associated with a redistribution and higher expression of CXCL13, the ligand of CXCR5. Interestingly, it has been shown that such CXCL13 staining is generally observed during late embryonic splenic vasculature, in which B cells are essentially immature.^73^ This spatial alteration of CXCL13 during SIV infection may contribute to the defect of Tfh cells in MLNs. Indeed, CXCR5 play a critical role in regulating the homing of memory CD4 T cells to the B-cell zones,^112,113^ and Tfh cells are subsequently lost in the absence of B-cell integration.^85^

Because MLNs are not only crucial for the immune response against pathogens, but are also essential to maintain commensal microbiota under immune control, and to maintain immune tolerance, this defect of Tfh cells reported in MLNs of SIV-infected RMs may contribute to microbial translocation and inflammation associated with AIDS. These results in monkeys may provide major advances for the understanding of mucosal immunology in humans. Therefore, strategies aiming to preserve Tfh cells can be beneficial to restore B-cell immunity during HIV and SIV infections.

## Materials and methods

### Ethics statement

All RMs were housed at Laval University, in accordance with the rules and regulations of the Canadian Council on Animal Care (http://www.ccac.ca). This protocol was approved by the Laval University Animal Protection Committee (Project number 106004). Animals were fed standard monkey chow diet, supplemented daily with fruit and vegetables, and water ad libitum. Social enrichment was delivered and overseen by a veterinary staff, and overall animal health was monitored daily. Animals showing significant signs of distress, disease, and weight loss were evaluated clinically and were humanely euthanized, using an overdose of barbiturates, according to the guidelines of the Veterinary Medical Association.

### Animal, viral inoculation, and sample collection

RMs (*Macaca mulatta*) seronegative for SIVmac, STLV-1 (Simian T Leukemia Virus type-1), SRV-1 (type D retrovirus) and herpes-B viruses were used in this study. RMs were infected intravenously with SIVmac251 virus (20 AID_50_). RMs were euthanized at different time points post-infection, covering both acute and chronic phases. Peripheral blood, spleen, axillary, and inguinal LNs, ileocolic including cisterna chyli, and colic MLNs were recovered for cellular analysis (as shown in supplementary Fig. 4). Cell numbers were calculated from LNs retrieved in each region (the totality of the MLNs were retrieved). For blood, a hemogram was elaborated using an Abaxis VetScan HM5 hematology instrument (Abaxis, CA). Tissues were not digested with collagenase or other proteases for cell isolation limiting side effects on the expression of cell surface markers.

### Immunophenotyping

Fresh cells were stained with a panel of monoclonal antibodies. The fluorochrome-conjugated antibodies used are provided in the Table S1. After lysing erythrocytes (Lysing buffer Pharm Lyse 10× BD Biosciences), 60,000 events corresponding to mononuclear cells were recorded in FACS Canto A (BD Bioscience). Intracellular Bcl-6, c-Maf, TCF1, Foxo1, KLF2, Eomes, and T-bet staining was performed after fixing and permeabilizing the cells with the FoxP3 staining buffer set (eBioscience), whereas pStat5 was detected after permeabilization using BD Cytofix^™^ Fixation Buffer. Analyses were performed using FlowJo software (Tree Star, Inc.).

### In vitro stimulation

MLN cells (1 x 10^6^) were cultured in 24-well plates pre-coated with purified anti-CD3 (0.1 μg/ml, Abcam) and with CD28 (0.25 μg/ml, Biolegend) in the presence of IL-6 (100 ng), and IL-21 (100 ng), and in the absence or presence of IL-27 (10 ng), IL-2 (10 ng), and IL-7 (10 ng). Four (4) days later, cells were stained with anti-PD1-PerCP-eFluor710, anti-CXCR5-PE, anti-CD4-APC-H7 and anti-CD20-PE-Cy7. The expressions of Foxo1, KLF2, STAT5, T-bet-, c-Maf- and TCF1 were assessed by flow cytometry.

### Immunofluorescence confocal microscopy of tissue sections

MLNs were embedded in optimal cutting temperature compound (OCT), sectioned at a 7.5 μm thickness and stored unfixed at −20 °C until use. Tissue sections were fixed in 4% PFA (15 min at room temperature) followed by acetone (20 min at −20 °C). Slides were submerged in blocking solution (5% normal horse serum, 0.3% triton X-100) for 1 h at room temperature. For panel 1 (CD4/CD20/CXCR5/PD1), sections were initially incubated overnight at 4 °C with a purified anti-CXCR5 antibody (kindly provided by the NIH Nonhuman Primate Reagent Resource) diluted in antibody dilution buffer (1% bovine serum albumin, 0.3% triton X- 100). The next day, sections were washed and incubated with a secondary antibody coupled with Alexa Fluor-488 (AF-488) diluted in antibody dilution buffer for 1 h at room temperature. After extensive washing, sections were incubated overnight at 4 °C with fluorochrome-coupled antibodies, including the CD4-BV421, CD20-eF615 and PD1-AF647. After washing, the slides were mounted with Prolong Gold antifade mounting medium. The procedure employed for Panel 2 (IL-21/CXCL13/CD20) was similar. Briefly, sections were initially incubated with a purified anti-IL21 antibody and a purified anti-CXCL13 antibody overnight, followed by two secondary antibodies, one coupled to AF-488 and one coupled to AF-546. After washing, samples were incubated overnight with the directly coupled antibody for CD20-eF660. S1 Table provides information regarding the antibodies used for tissue immunofluorescence. Sections were imaged in a Zeiss LSM 710 confocal microscope. Tiled Z-stacks were acquired with a 20× objective and stitched using the Image J stitching plugin.^114^ Average intensity projections were obtained from the stitched tiles using built-in Image J tools.

### In situ hybridization

Productively infected cells (SIV^+^ RNA cells) were assessed in LNs by in situ hybridization, as previously described.^16^ Infected cells were detected and counted in the paracortical zone on a minimum of three sections using a Nikon-FXA microscope. A ^35^S-labeled RNA probe derived from the SIVmac *nef* gene was used. To enhance probe penetration into the tissue sections, the ^35^S-labeled RNA was subjected to mild alkaline hydrolysis to obtain a majority of fragments in the 150- to 200-nucleotide range. The number of positive cells was then divided by the surface of the entire LN section, and the results were expressed as the number of positive cells per 2-mm^2^ section. The mean count was calculated for three slides of the same LNs obtained in a blinded fashion by two different investigators.

### Statistical analysis

Statistics were performed with GraphPad Prism 5 software. The nonparametric Mann–Whitney test and Wilcoxon tests were used, as indicated.

## Supplementary information

Supplementary Information

Supplementary Table 1
