# Supplementary material for: Mucosal T follicular helper cells in SIV-infected rhesus macaques: contributing role of IL-27
Source: Mucosal Immunol. 2019 May 21;12(4):1038–54. doi: 10.1038/s41385-019-0174-0 (PMC7746526; doi:10.1038/s41385-019-0174-0)
Supplement: Supplementary file 1 — Supplementary Information [file 41385_2019_174_MOESM1_ESM.pdf]

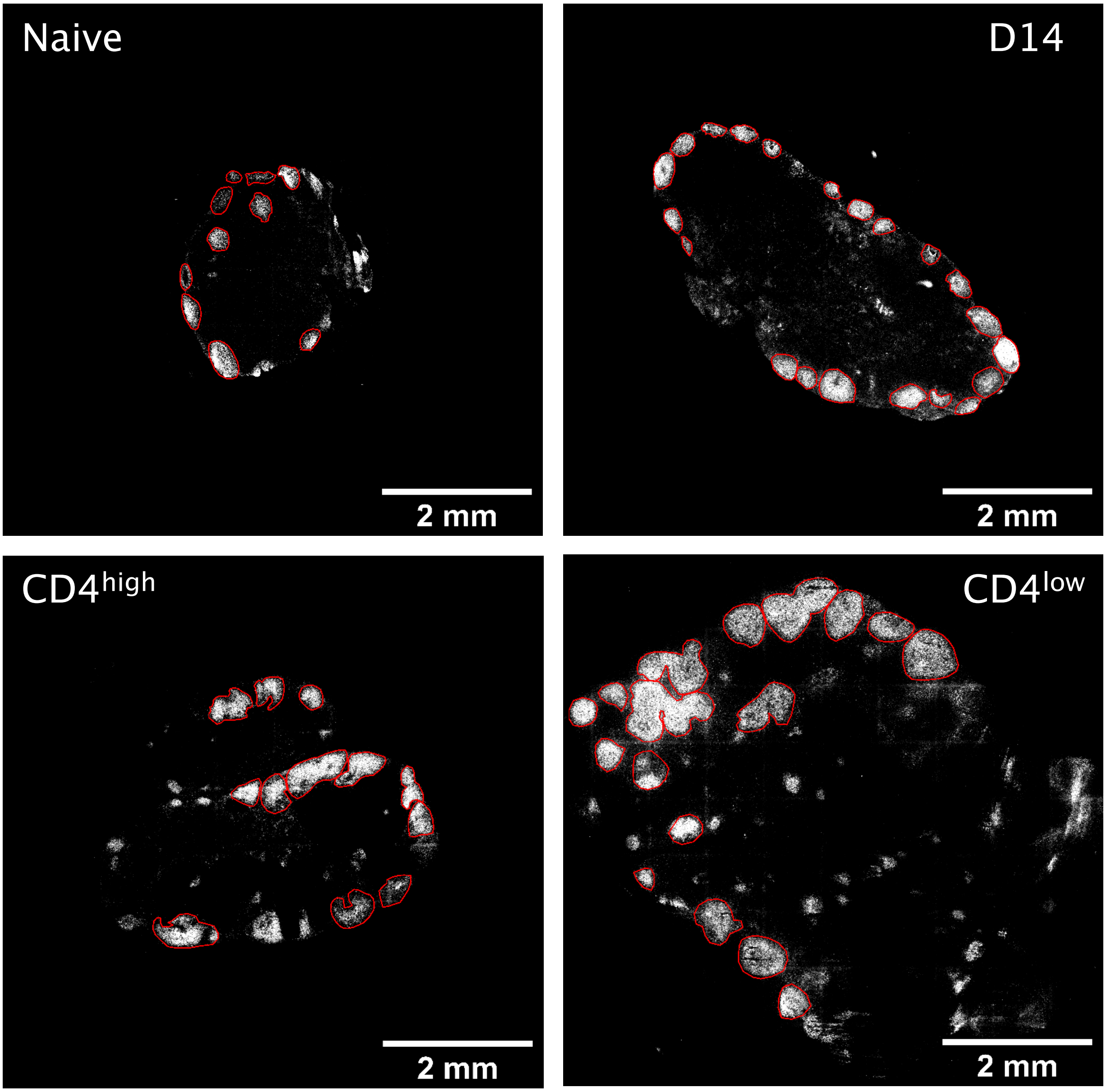

MLN sections showing B cell follicles in which the contours have been defined for B cell area quantification using the Image J software. Herein four RMs are shown.

Supplementary figure 1

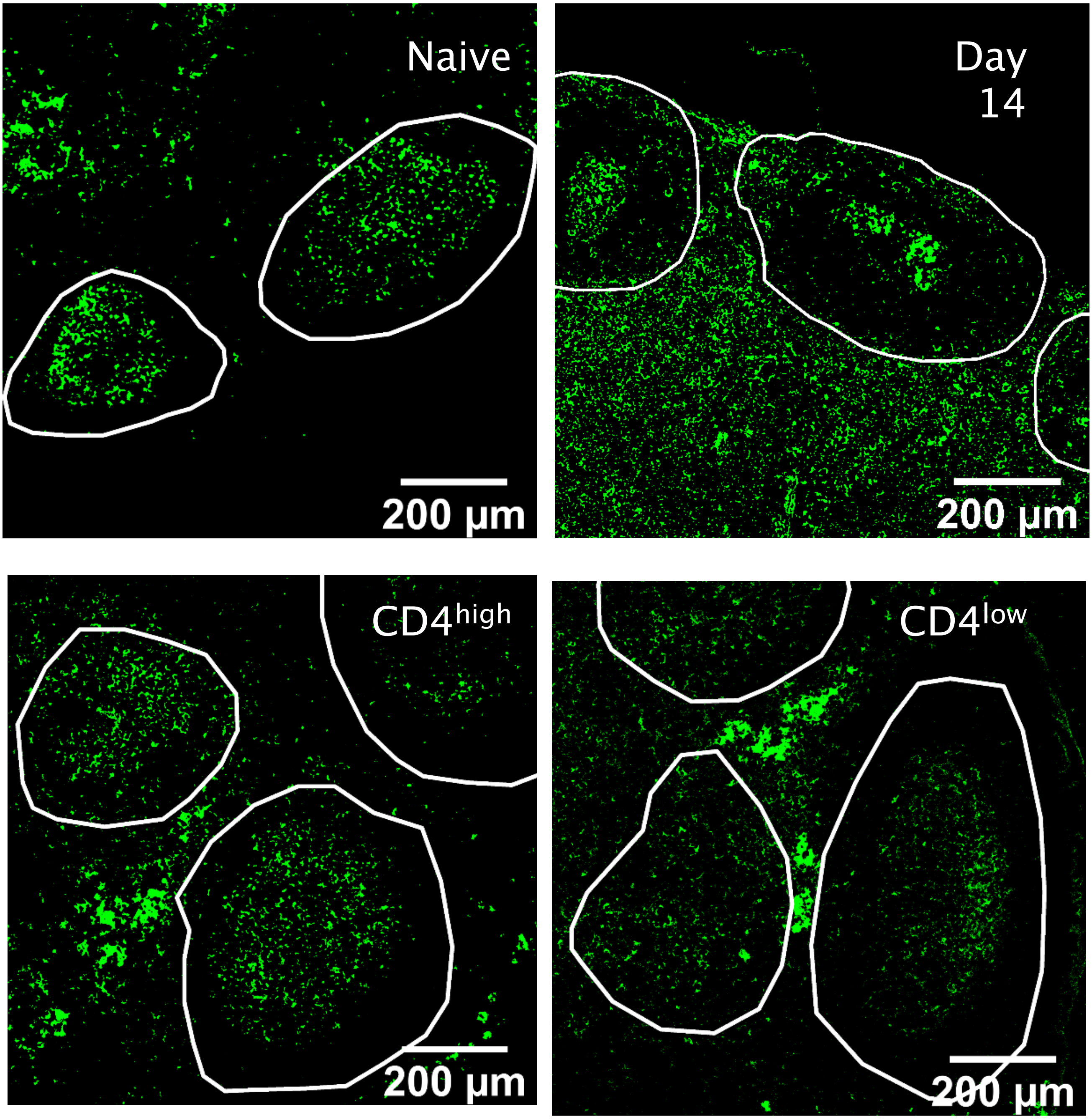

MLN sections showing IL-21 staining. Contours of B cell area are indicated to quantify IL-21 staining using the Image J software. Herein four RMs are shown.

Supplementary figure 2

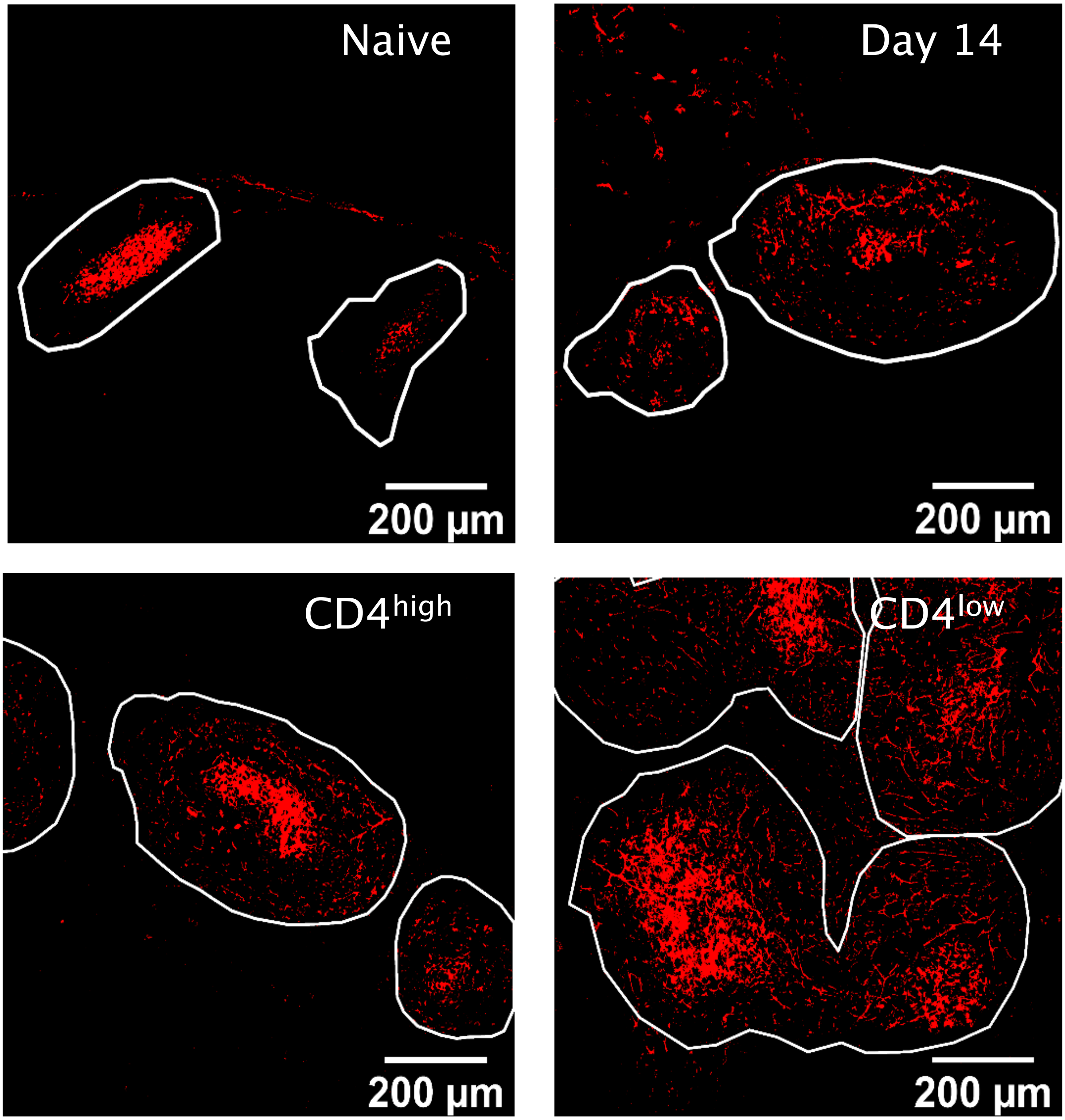

MLN sections showing CXCL13 staining. Contours of B cell area are indicated to quantify CXCL13 staining using the Image J software (kurtosis index). Herein four RMs are shown.

Supplementary figure 3

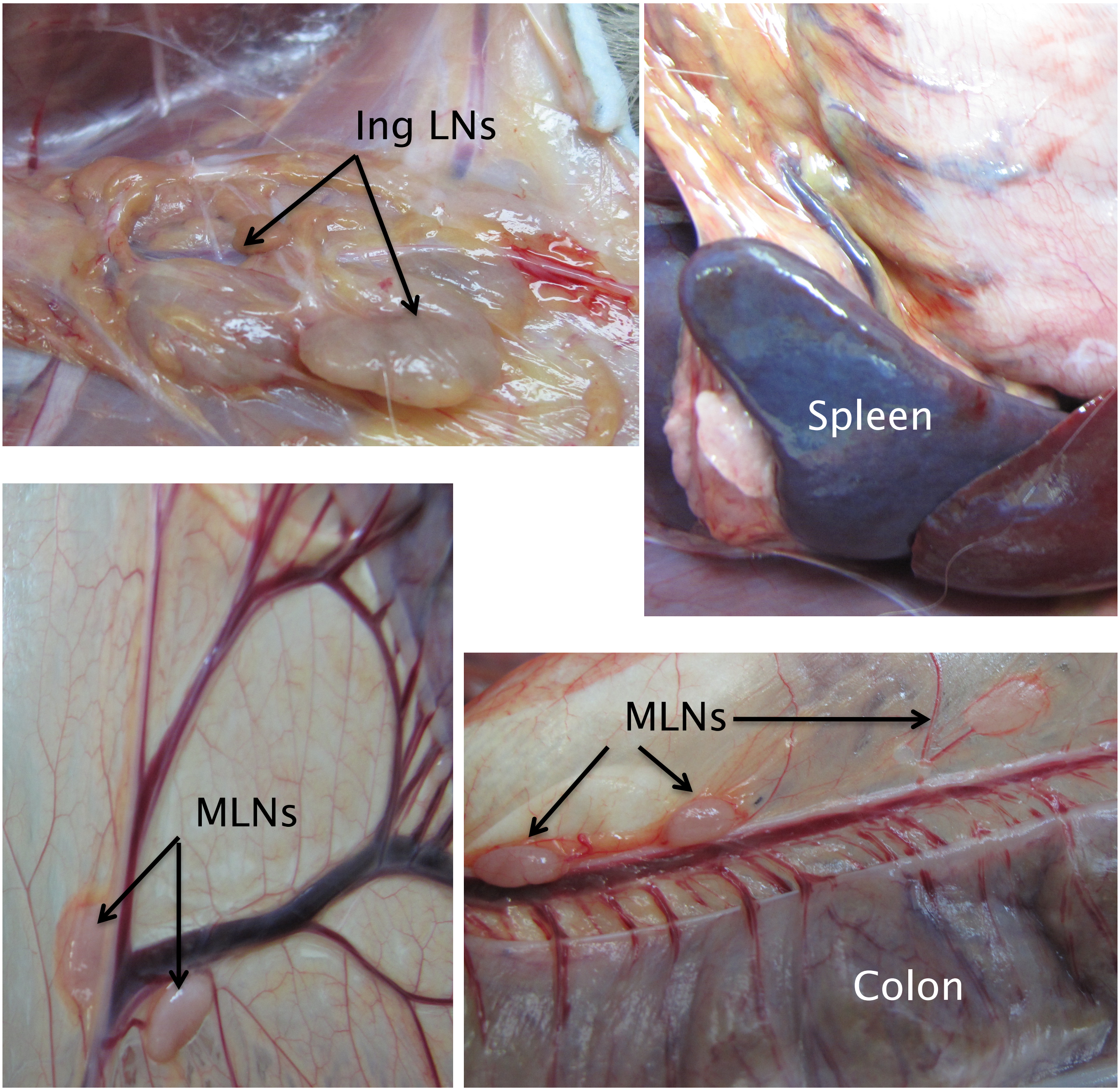

Lymphoid tissues in RMs: Representative pictures of peripheral inguinal (Ing) LNs, spleen and mesenteric (MLNs) LNs along the mesentery and the colon (large intestine).
