# Supplementary material for: Mucosal T follicular helper cells in SIV-infected rhesus macaques: contributing role of IL-27
Source: Mucosal Immunol. 2019 May 21;12(4):1038–54. doi: 10.1038/s41385-019-0174-0 (PMC7746526; doi:10.1038/s41385-019-0174-0)
Supplement: Supplementary file 2 — Supplementary Table 1 [file 41385_2019_174_MOESM2_ESM.docx]

**Supplementary Table 1**

| **Antibody** | **Fluorochrome** | **Clone** | **Manufacturer** |
| --- | --- | --- | --- |
| **Flow cytometry** | | | |
| α-CD62L | FITC | SK11 | BD Biosciences |
| α-CXCR5 | PE | 87.1 | eBiosciences |
| α-CXCR5 | PE | MU5UBEE | eBiosciences |
| α-PD-1 | PerCP-eFluor710 | J105 | eBiosciecnes |
| α-CD45RA | PE-Cy7 | 5H9 | BD Biosciences |
| α-CD4 | APC | L200 | BD Biosciences |
| α-CD4 | APC-H7 | L200 | BD Biosciences |
| α-CD3 | APC-Cy7 | SP34-2 | BD Biosciences |
| α-c-Maf | eFluor660 | Sym0F1 | eBiosciecnes |
| α-CD21 | FITC | B-ly4 | BD Biosciences |
| α-CD27 | PE | M-T271 | BD Biosciences |
| α-CD20 | PE-Cy7 | 2H7 | BD Biosciences |
| α-KLF2 | Alexa Fluor488 | bs-2772R-A488 | Bioss Antibodies |
| α-Foxo1 | Alexa Fluor488 | bs-2537R-A488 | Bioss Antibodies |
| α-T-bet | eFluor660 | eBio4B10 | eBiosciecnes |
| α-Eomes | FITC | WD1928 | eBiosciecnes |
| α-Phospho-Stat5 | Alexa Fluor 488 | C71E5 | Cell Signaling |
| α-TCF1 | Alexa Fluor 647 | C63D9 | Cell Signaling |
| CD3 | - | FN-18 | Abcam |
| CD28 | - | CD28.2 | Biolegend |
| **Tissue immunofluorescence** | | | |
| α-CXCR5 | Purified | 710D82.1 | NHP Reagent Resource |
| α-CD4 | BV421 | OKT4 | Biolegend |
| α-PD-1 | AF647 | EH12.2H7 | Biolegend |
| α-CXCL13 | Purified | Polyclonal | R&D system |
| α-IL21 | Purified | Polyclonal | AbD Serotec |
| α-CD20 | eF660 | L26 | BD Biosciences |
| α-CD20 | eF615 | L26 | eBioscience |
